# Investigating visual expertise in sculpture: A methodological approach using eye tracking

**DOI:** 10.16910/jemr.15.2.5

**Published:** 2022-06-30

**Authors:** Isabell Stein, Helen Jossberger, Hans Gruber

**Affiliations:** University of Regensburg, Germany; University of Turku, Finland

**Keywords:** Art perception, Mobile eye tracking, Sculpture, Visual expertise

## Abstract

Research on visual expertise has progressed significantly due to the availability of eye tracking tools. However, attempts to bring together research on expertise and eye tracking methodology provoke several challenges, because visual information processes should be studied in authentic and domain-specific environments. Among the barriers to designing appropriate research are the proper definition of levels of expertise, the tension between internal (experimental control) and external (authentic environments) validity, and the appropriate methodology to study eye movements in a three-dimensional environment. This exploratory study aims to address these challenges and to provide an adequate research setting by investigating visual expertise in sculpting. Eye movements and gaze patterns of 20 participants were investigated while looking at two sculptures in a museum. The participants were assigned to four different groups based on their level of expertise (laypersons, novices, semi-experts, experts). Using mobile eye tracking, the following parameters were measured: number of fixations, duration of fixation, dwell time in relevant areas, and revisits in relevant areas. Moreover, scan paths were analysed using the eyenalysis approach. Conclusions are drawn on both the nature of visual expertise in sculpting and the potential (and limitations) of empirical designs that aim to investigate expertise in authentic environments.

## Research on visual expertise

Human expertise is of considerable societal interest because excellent professional performance influences everyone’s daily lives, cultural achievements, the quality of the health system or the economic success of organisations. Research on expertise has made significant progress since the information-processing paradigm (36) began to shape psychological and educational theories of human performance and professional learning.

The main contributions of research on expertise focus on cognitive adaptations during long and deliberate practice periods as well as the execution of professional work in authentic workplace environments (6). Therefore, expertise is considered highly domain-specific. For some decades, research on expertise has investigated cognitive structures and processes, mainly considering memory processes and those of knowledge acquisition, storage and retrieval. Indeed, evidence suggests that it is a common characteristic of expertise development across different domains that the core process is knowledge restructuring through the processing of authentic cases while deliberately experiencing professional situations (3).

Although some studies from the 1960s (17; 35) have found that processes of knowledge restructuring are closely related to visual and perceptual processes, since experts are clearly able to quickly scan complex domain-specific professional stimuli and to extract relevant information for further processing, the role of visual expertise has been negligible in research on expertise. One reason was that adequate measurement of these visual processes was barely possible. Lesgold et al. (23) were one of the first who conducted eye-tracking studies to investigate visual expertise in the complex field of medicine. Subsequently, the development of eye tracking tools in the last 10 to 15 years has changed this situation considerably. Psychological and educational studies are now widely available, and this is even true in authentic professional situations thanks to the advent of mobile eye tracking facilities (19).

Jarodzka et al. (15) have identified several educational research topics that could be emphasised, especially the investigation of visual expertise in professional domains with a strong visual component in professional actions. In their introduction to a special issue devoted to the field of teaching, Jarodzka et al. (16) describe the research on visual expertise of teachers as challenging because of the complexity in the real-life scenario in a classroom. They point out that the absorption and interpretation of information occurs to a large extent through visual perception and that eye tracking can help to visualize and investigate these processes. Similarly challenging is it to investigate visual expertise of artists in the complex scenario of art museums.

Boshuizen et al. (3) have explicitly analysed characteristics of professional action in authentic workplace environments that shape differences and commonalities across domains. They found that such characteristics play an important role in the research design applied, based on such key questions as: “Who is considered to be an expert in the domain?”, “What are important professional tasks in the domain?”, “How is professional learning organised in the domain?”, “How clear is canonical knowledge defined in the domain?”, and so on. They found that empirical studies conducted in different domains often implicitly answer the questions differently, and it is a major challenge to address these research assumptions. Here, we argue that it is particularly difficult to do so in artistic domains, and in fine art in particular.

## Investigating attentional processes by eye tracking and visual expertise in fine art

The reception of works of art plays an important role in the life of an artist, even if the focus is usually more on practical artistic work. Reception and creative work go hand in hand. The exchange with colleagues, but also looking at the works of other artists help to break new ground in the own creative process. Therefore, it is important to examine both areas, the practical work of a sculptor (31) but also the reception of sculptures by the sculptor, which is the focus of the present study.

One of the pioneers in tracking eye movements as indicators of visual attentional processes was Buswell (4) presenting a wide variety of data over different areas like reading, picture viewing and perception of art. Yarbus (42) provided one of the best-known studies that investigated knowledge-based differences in visual processes in the arts. He found that eye movements while looking at a picture vary dramatically if different information about the semantic content of the picture is presented in advance. Lawrence Stark and associates (30; 34; 43) put forward the idea of scanpaths as a temporally ordered sequence of fixations controlled by the mental models of the viewer in a top-down processing strategy. Rudolf Groner and associates (10; 26) extended Lawrence Stark’s concept of scanpaths to two different classes of scanning processes: local and global scanpaths. Local scanpaths are assumed to be processes on the perceptual level operating bottom up on a narrow time scale (i.e. releasing the next saccade), while global scanpaths are assumed to be top-down driven by cognitive processes and operate on an extended time scale (i.e., releasing a group of saccades controlled by concepts and expectations).

Looking at artwork is performed systematically in museums (27; 33). For most people, visiting an art museum is an exciting activity; artwork is perceived, analysed and interpreted. Depending on their prior knowledge and experiences, individuals view works of art differently. More specifically, research has indicated that experts tend to have a more global viewing pattern than less experienced persons (29; 38), as well as a higher global/local ratio (38; 44).

Various models describe the reception of two-dimensional artwork (2; 18; 28). In these models, the exploration process is often divided into an initial global phase and a subsequent local phase. Reception begins with an exploration phase in which short fixations are carried out. The second phase is characterised by longer fixations as the viewer takes a closer look at the details of the artwork (this global/local shift is also referred to as diverse/specific or ambient/focal). Kozbelt and Ostrofsky (21) have summarised the state-of-the-art about expertise in drawing, while Chamberlain et al. (5) have presented an update on research on visual expertise in the arts.

Surprisingly, much less is known about three-dimensional settings like sculpture, even though sculpture has been a core activity in fine art since the ancient world. Recent work by Puppe et al. (32) has outlined certain peculiarities of expertise development in sculpture. Puppe et al. (31) investigated the eye movements of professional sculptors and sculpture students when looking at a model and then creating their own artwork. They found evidence that individuals with different levels of expertise differ in their visual processes across a multitude of processes; as a consequence, they use the model quite differently in their own artistic work.

The nature of looking activities may thus be different with three-dimensional stimuli like sculptures compared to two-dimensional stimuli like painting (24). By walking around a sculpture, for example, one can always adopt a new point of view and thus explore the work of art with a new gaze. As a result, global and local viewing processes could alternate repeatedly throughout the viewing process. Indeed, it has been argued in art education that the development of differentiated spatial perception mainly takes place through such systematic alternation. The process of looking at sculptures may be considered as a repetitive jumping back and forth between the work’s general ‘global’ form and its individual ‘local’ details. In turn, the global/local ratio may be somewhat different compared to looking at two-dimensional art (e.g., paintings), and it is plausible that individuals with different levels of expertise systematically look differently at sculpture, as is the case with the perception of two-dimensional artworks (38; 44).

It might be assumed that with an increase in the level of expertise, an increased number of switches would be performed between these two modes of perception (i.e., global and local viewing). However, with the exception of Wiseman et al. (40), which is conceptual rather than empirical, we are unaware of any studies that have systematically investigated how visual processing of three-dimensional artwork is performed by individuals with different levels of expertise. Thus, our study may constitute pioneering work in the processing of three-dimensional stimuli in an authentic setting within the domain of visual arts, namely sculpture.

As explained above, considerable challenges are associated with such an attempt. First, the research on expertise has hardly touched on the domain of sculpture, meaning that the questions addressed by Boshuizen et al. (3) remain unanswered: How can different levels of expertise plausibly be defined and differentiated? What are the “natural” activities of visually studying sculptures? Both aspects are to be considered in this study, which applies mobile eye tracking technology in an authentic environment (museum exhibition) to analyse visual processes while looking at sculptures.

In the domain of sculpting, artists or experts may differ qualitatively from laypersons since they are focused on other aspects of the artwork. For example, the artists’ reduced fixation time on “recognisable” objects depicted in studies by Nodine et al. (29) and Vogt and Magnussen (38) may indicate that artists tend to pay more attention to the compositional and structural characteristics of a work; investigations with art history experts yielded similar results (12; 39). Thus, it is plausible that the locations on which people fixate may differ in a three-dimensional work of art and that these differences correspond to the expertise level. Although eye movements have an individual structure, differences in eye movement patterns should be larger between the expertise groups than within these groups. So far, this assumption has not yet been investigated empirically.

## Aim and research questions

The main purpose of this study is to provide an exploratory understanding of the potential and limitations of an approach that uses eye tracking measures to better understand how participants with various levels of expertise process visual information while looking at sculptures. We wanted to find out how methodological innovations investigating eye movements and gaze patterns during the reception of sculptures can be designed to contribute to the understanding of participants who differ in their level of expertise in sculpture. The state-of-the-art suggests the following research questions:

Research question 1: Is a higher level of expertise associated with a larger global/local ratio?

Research question 2: Is a higher level of expertise associated with a higher frequency of switching between global and local viewing?

Research question 3: Do the locations of the fixations and the fixation durations differ more between participants with increasing divergence of expertise level?

Research question 4: Is a higher level of expertise associated with an increased number of fixated basic features?

Research question 5: Is a higher level of expertise associated with an increased percentage of fixations on basic features?

Research question 6: Is a higher level of expertise associated with an increased number of revisits per minute to basic features?

## Method

### Design

This study used a 4 × 2 design with repeated measures on the second factor. Between-factor was “level of expertise” (four levels: layperson, novice, semi-expert, expert). Within-factor was the stimulus (two levels = two sculptures: Moses, Daphne).

### Participants

Participants were museum visitors who were willing to wear mobile eye trackers during their visits and who had filled in a short questionnaire to determine their level of expertise in sculpting.

From a total of 36 participants, due to a high failure rate (poor calibration due to dry eyes, contact lenses, glasses, mascara, etc.), data from 20 participants were included in the analysis (N = 20). Twelve females and eight males aged between 23 and 75 participated.

Each of the 20 participants was assigned to one of the groups defined by their level of expertise in the reception of artwork but also in the creation of artwork: five laypersons (no arts or arts education background; mean age = 38.2 years, SD = 20.4 years), five novices (Bachelor’s students of Art Education; mean age = 32.1 years, SD = 15.9 years), five semi-experts (Master’s students or graduates of Art or of Art Education; mean age = 33.9 years, SD = 17.7 years), five experts (professional sculptors with at least 10 years of experience in sculpting; mean age = 41.6 years, SD = 15.8 years).

The experts had been working intensively in the field of sculpture for around 14, 15, 24, 25 and 29 years, respectively (see Table 1). On average, they had 21.4 years of experience in sculpture. All of them were artists who regularly exhibit their own works to the public and have already taken part in artistic competitions, and all were members of the relevant professional association of fine art. Moreover, the artists regularly visit exhibitions and are in lively exchange with other artists.

According to guidelines from research on expertise (3; 7), group assignment was based on the participants’ experience with sculpture: no experience at all = layperson; less than five years of experience = novice; between 5 and 10 years of experience = semi-expert; more than 10 years of experience = expert (see Table 1).

All participants had normal or corrected-to-normal vision.

**Table 1.** Classification of experience in the domain of sculpting in years into expertise levels by thresholds.

Expertise level	Lay-persons	Novices	Semi-experts	Experts
n	5	5	5	5
Experience in the domain of sculpting in years	0	0.25-4.5	6-9	14-29
Thresholds in years	0	<5	>5<10	>10

### Materials

The questionnaire comprised questions about the sculpting activities of the participants, including their experience (length, intensity) in their own artistic work, academic and artistic careers, as well as reception activities (i.e., frequencies of visit to exhibitions, to artists’ studios).

Mobile eye tracking glasses (SMI GmbH, Teltow / Berlin, Germany) with a temporal resolution of 30 Hz were used. The objects of investigation were two sculptures that were regularly exhibited in a well-known and internationally prestigious art museum, the Kunstforum Ostdeutsche Galerie in Regensburg, Germany (see Appendix, “Artworks/Stimuli”): ‘Untitled (Moses)’ (hereafter Moses), a bronze sculpture measuring 38.9 × 17.2 × 19.9 cm, and ‘Great Daphne’ (hereafter Daphne), a bronze sculpture on a pedestal of dark grey shell limestone (144.0 × 29.5 × 25.5 cm).

Both sculptures were exhibited in a free space in the museum so that visitors could walk around and view them from all sides. The walk through the museum suggested that visitors looked first at Moses and then at Daphne; all participants adhered to this viewing order. The choice fell on these two sculptures, as both were some of the few in the exhibition that could be viewed from all sides. Both sculptures are very complex, the surface texture and the associated evaluation of basic features, which were to be investigated with expert defined AOIs, also led to the selection. Besides these similarities, there are also interesting differences between the sculptures. The sculpture Daphne is more realistic with many small details such as leaves whereas Moses is more abstract and roughly worked. Hence, surface structures can be perceived rather than representational details. These differences were important to reduce stimuli related influences, such as personal preferences.

### Procedure

The study took place among the daily activities in the museum in which the two sculptures were exhibited. It was an open-ended (i.e., without time restriction) and free exploration task, whereby participants were asked to look at the sculptures for as long as and in whichever way they wanted. No explicit instruction was given to support as natural as possible a setting in the museum.

### Analysis

BeGaze Version 3.4.2 (SMI GmbH, Teltow / Berlin, Germany) was used to analyse the eye tracking data.

**Figure 1.**
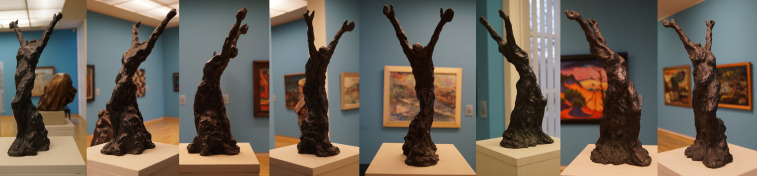
Eight reference images of different directions of the sculpture Moses by Kroner, 1919

Informal information from art history works suggests that a total of eight different views provides the best means of capturing a sculpture in its entirety. Walking around the sculpture, this means that an angle of 45° distinguishes each perspective. Based on this assumption, eight reference images of each sculpture were taken for the mapping process (an example for one of the sculptures is depicted in Figure 1). All analyses of the mapping of areas of interest between perspectives had to be coded manually. For the calculations, the fixations of all reference images were summarised to include the entire observation in the analysis. Eye movement data were analysed with MATLAB.

Given the free observation, the participants looked at the sculptures for different lengths of time. To minimise the influence of the length of observation time, global local ratio, switching between global and local viewing and revisits per minute are not absolute values but relative values over time. Thus, for example, switching between global and local viewing is not simply given in terms of the number of switches, but in switches per second.

To address research questions 1 and 2, the duration of fixation was used as an indicator to differentiate between global and local viewing. Based on research on two-dimensional stimuli (29), the mean fixation duration (M = 187 ms) was used as a threshold. Fixations longer than 187 ms were used as indicators for a local viewing process, while fixations shorter than 187 ms indicated a more global viewing strategy.

To address research question 3, a measurement was used to calculate the differences between scan paths of pairs of participants regarding the location and duration of fixations. For this purpose, the “eyenalysis” method was applied according to Mathôt et al. (25).

**(1):**
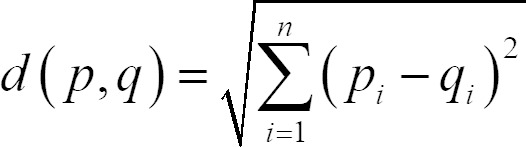


**(2):**
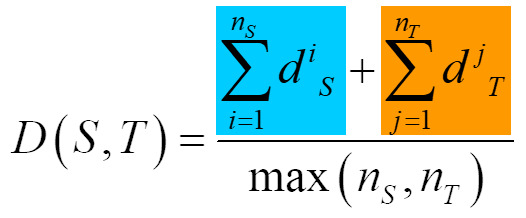


representing the mapping between a point p in S and a point q in T, associated with a distance, d(p,q), the Euclidean distance between p and q, summed over n which is the number of dimensions, and p_i_ and q_i_ are the i-th dimension of p and q, respectively.

**Figure 2.**
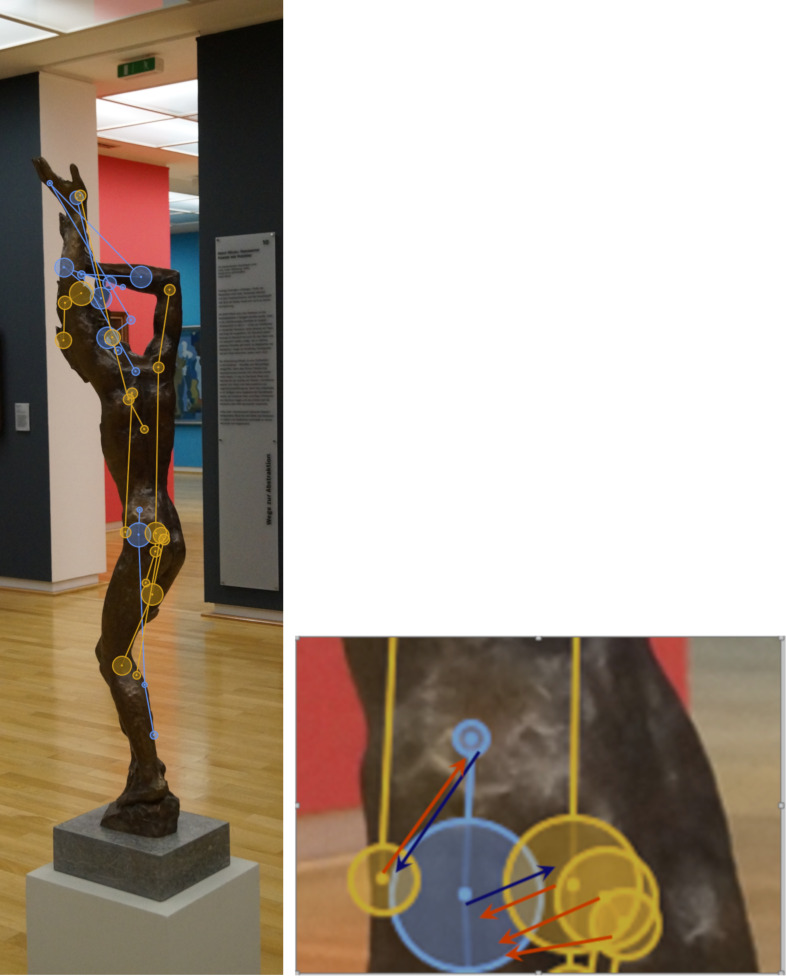
Example for the double mapping of the fixations of participants Blue and Orange according to the eyenalysis method on the sculpture Daphne (Sintenis, 1930/1991).

According to the eyenalysis method of Mathôt et al. (25), the distance between two participants S and T – we call them Blue and Orange – was calculated (see Figure 2).

For each fixation of participant “Blue”, the closest fixation of participant “Orange” was identified and the distance between both fixations was calculated, and vice versa. By applying this “double mapping”, it was possible to include all fixations, irrespective of whether Blue and Orange differed in the number of fixations. The geometric distance of the fixation pairs (see arrows in Figure 2) was also calculated.

For each fixation, three dimensions were considered: x-coordinate, y-coordinate and fixation duration. The fixation time represented the third dimension of the coordinate system in which the distance was calculated. Since the x and y coordinates were in the unit px (pixels), a conversion factor was introduced to include the fixation time (in ms). This step was performed so that the duration of the longest fixation to a sculpture corresponded to the height of the respective sculpture. Thus, the largest expansion on the axis for the fixation duration corresponded to the largest expansion on the y-axis. For Moses, this resulted in a conversion factor of 1.097 px per ms; for Daphne, a conversion factor of 1.731 px per ms was obtained. The sum of both directions of the comparison (double mapping) were then divided by the respective larger number of fixations of the two participants.

To ensure that all reference images were included in the present study, the eyenalysis method (25) was extended using the following procedure. The distance of two data sets was calculated for each reference image. Subsequently, the mean value was calculated. This served as a measure for similarity between two eye tracking data sets, which we refer to as “distance”. In this way, the distances between all participants were calculated. To investigate the effect of the divergence of the expertise level on these distances, the distances were divided into four groups according to the divergence of the levels of expertise (see Table 2). The group without divergence in level of expertise was given the value 0, since there is no divergence in expertise level. For example, the divergence in the expertise level between laypersons and laypersons, but also between experts and experts, is 0. By their very nature, group sizes vary in this process, as different numbers of pairwise comparisons are made within the groups. For example, if one compares 5 laymen within the group, one receives 10 comparisons. However, if one compares 5 laymen with 5 novices, one receives 25 comparisons.

**Table 2.** Allocation of the distances into four groups with divergent expertise levels.

Divergence of expertise level							
0 (N=40)		1 (N=75)		2 (N=50)		3 (N=25)	
Lay/Lay	N=10	Lay/Nov	N=25	Lay/Sem	N=25	Lay/Exp	N=25
Nov/Nov	N=10	Nov/Sem	N=25	Nov/Exp	N=25		
Sem/Sem	N=10	Sem/Exp	N=25				
Exp/Exp	N=10						

**Figure 3.**
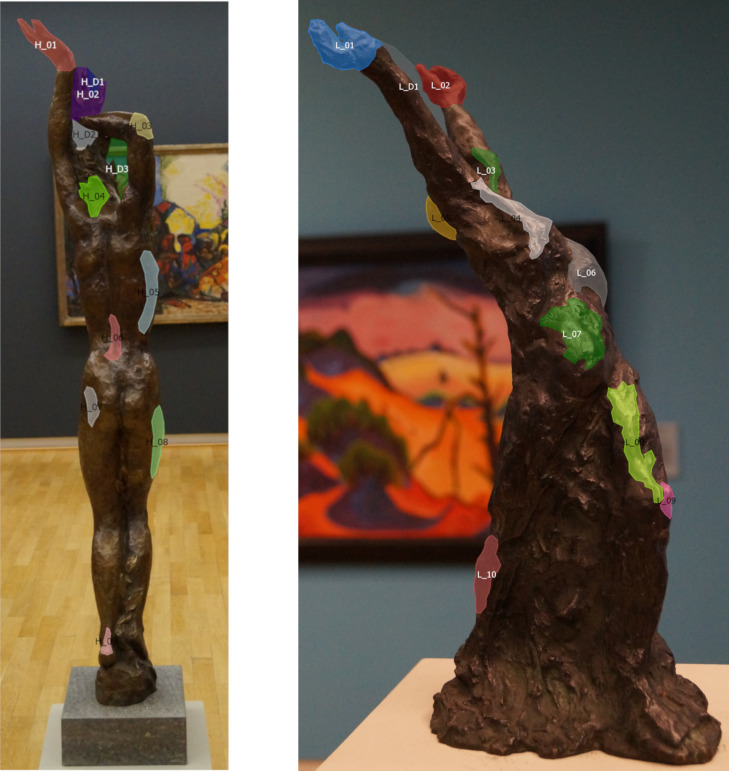
Expert defined AOIs also known as basic features on Daphne (on the left, Sintenis, 1930/1991) and Moses (on the right, Kroner, 1919) exemplary on one reference image.

To address research questions 4, 5, and 6, basic features were determined a priori as expert-defined areas of interest (see Figure 3). The expert was not part of the sample; she is professor for art education and a professional sculptor with more than ten years of professional experience.

To address the revisits of those basic features (research question 6), revisits were defined as follows: a basic feature was fixated, then at least one fixation took place outside this basic feature, and then another fixation was performed within this basic feature. A long dwelling of the gaze within one basic feature was not counted as revisit if the basic feature was not left in the meantime.

Due to the small sample size and the exploratory nature of the research questions, only descriptive data are presented. We investigated the extent to which trends could be observed or whether the groups of participants displayed no remarkable differences.

## Results

Although participants could explore the sculptures without time restriction and hence differed in inspection time, the inspection across both sculptures did not differ significantly between levels of expertise. In addition, the double mapping procedure reduced the impact of inspection time.

Research question 1: Is a higher level of expertise associated with a larger global/local ratio?

Based on the threshold of 187 ms, the mean fixation duration was smaller than in previous studies with two-dimensional stimuli. The global/local ratio was calculated individually for each participant, and the respective mean value was calculated for each group (see Table 3).

**Table 3.** Mean values and standard deviations of the global/local ratio, by expertise level and by sculpture.

Expertise level	Moses		Daphne	
	M	SD	M	SD
Laypersons	2.00	1.52	1.54	0.59
Novices	1.73	0.94	2.09	1.39
Semi-experts	2.14	2.21	1.48	0.73
Experts	2.09	2.02	1.70	1.14
Total	1.99	1.61	1.70	0.96

When looking at Moses, the semi-experts had the highest global/local ratio. For Daphne, however, the novices had the highest global/local ratio. For both sculptures, the laypersons had the second lowest global/local ratio and the experts the second highest global/local ratio. Overall, Moses triggered a higher global/local ratio than Daphne. In general, the differences between the levels of expertise were negligible. Therefore, research question 1 was not supported.

Research question 2: Is a higher level of expertise associated with a higher frequency of switching between global and local viewing?

Again, the threshold of 187 ms was used to distinguish global and local perception. The number of switches between global and local viewing was counted for each participant and divided by the duration of their inspection time. Here, the results differ for the two sculptures (see Table 4).

**Table 4.** Mean values and standard deviations of the frequency of switching between global and local viewing per second, by expertise level and sculpture.

Expertise level	Moses				Daphne			
	M	SD		M	SD
Laypersons	0.50 Hz	0.35 Hz		0.53 Hz	0.34 Hz
Novices	0.59 Hz	0.36 Hz		0.70 Hz	0.35 Hz
Semi-experts	0.64 Hz	0.33 Hz		0.52 Hz	0.13 Hz
Experts	0.60 Hz	0.32 Hz		0.71 Hz	0.30 Hz
Total	0.58 Hz	0.38 Hz		0.61 Hz	0.29 Hz

For Moses, the semi-experts switched most frequently between the two perception modes. In the case of Daphne, the experts switched most frequently between global and local viewing. The experts were also in second place after the semi-experts for Moses. The lowest frequency of switching between global and local viewing was found in the laypersons’ examination of Moses and in the semi-experts’ examination of Daphne. In general, the differences between the levels of expertise were negligible. The data appear not to support research question 2.

Research question 3: Do the locations of the fixations and the fixation durations differ more between participants with increasing divergence of expertise level?

Concerning the distances of fixation locations, the matrices in Tables 5 and 6 show the mean distance between all pairs of participants from the expertise groups specified, while mean distances within the same expertise level are given on the main diagonal. Table 5 reveals that the largest mean distance in the reception of Moses was found between laypersons and semi-experts. The smallest distance for Moses was among the laypersons.

**Table 5.** Mean values and standard deviations of the distances between the expert groups in the sculpture Moses.

		Lay-persons	Novices	Semi-experts	Experts
Lay-persons	M	1372	8479	9434	7660
	(SD)	(832)	(9719)	(5536)	(6892)
Novices	M		6410	6632	5246
	(SD)		(4778)	(6692)	(5676)
Semi-experts	M			8483	6365
	(SD)			(7410)	(7413)
Experts	M				4961
	(SD)				(4499)

**Table 6.** Mean values and standard deviations of the distances between the expert groups in the sculpture Daphne.

		Lay-persons	Novices	Semi-experts	Experts
Lay-persons	M	7073	10423	10921	16898
	(SD)	(4707)	(9843)	(13428)	(17849)
Novices	M		11235	7520	11496
	(SD)		(7911)	(10274)	(14055)
Semi-experts	M			2089	4937
	(SD)			(1436)	(5136)
Experts	M				7864
	(SD)				(8228)

**Figure 4.**
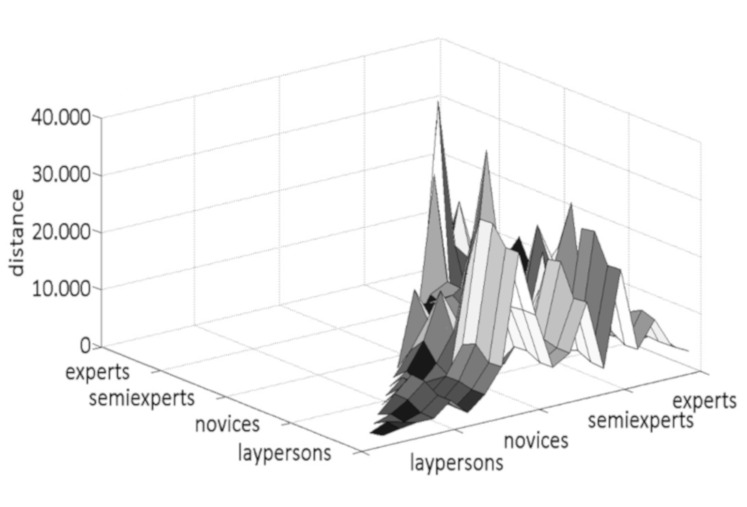
Distances of the fixation locations between all participants on the sculpture Moses.

**Figure 5.**
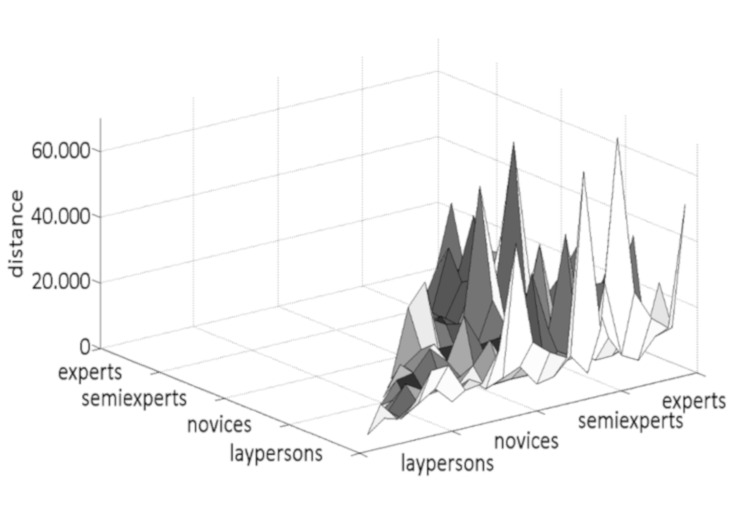
Distances of the fixation locations between all participants on the sculpture Daphne.

Figure 4 and 5 show the distances between all test subjects as single values rather than mean values. The graphs drop into a valley on the left, where the participants were compared with themselves; this zero-line was removed from the subsequent analysis. The distances for Moses show a flat area within the layperson group (see Figure 4). The second lowest distance was observed within the group of experts.

A similar result could be observed for the distances for Daphne (see Table 6 and Figure 5). Again, there is a flat area among the laypersons, whereas the smallest distance this time is among the semi-experts. The largest distance can be seen between the laypersons and the experts.

To address research question 3, the relationship between the divergence in the levels of expertise and the distances according to the eyenalysis method (25) were investigated. According to this method, the calculation of the distances of the eye movement data cannot be calculated for each subject individually, but only between subjects. A strict comparison between the laymen, experts, semi-experts, and novices, as it was conducted for the other research questions, is not possible. Therefore, as mentioned above, all results were grouped according to the divergence of the expertise level (compare with Table 2). The groupings are shown in Tables 7 and 8.

**Table 7.** Mean values of the distances grouped according to the divergence of the expertise level at the sculpture Moses.

Divergence of expertise level							
0		1		2		3	
Subjects	M	Subjects	M	Subjects	M	Subjects	M
Lay/Lay	1372	Lay/Nov	8479	Lay/Sem	9434	Lay/Exp	7660
Nov/Nov	6410	Nov/Sem	6632	Nov/Exp	5246		
Sem/Sem	8483	Sem/Exp	6365				
Exp/Exp	4961						

**Table 8.** Mean values of the distances grouped according to the divergence of the expertise level at the sculpture Daphne.

Divergence of expertise level							
0		1		2		3	
Subjects	M	Subjects	M	Subjects	M	Subjects	M
Lay/Lay	7073	Lay/Nov	10423	Lay/Sem	10921	Lay/Exp	16898
Nov/Nov	11235	Nov/Sem	7520	Nov/Exp	11496		
Sem/Sem	2089	Sem/Exp	4937				
Exp/Exp	7864						

To examine the factor of divergence on reception, mean values and standard deviations of the whole groups (0-3) were calculated and are presented in Table 9.

**Table 9.** Mean values and standard deviations of the distances with different divergence of the expertise level.

Divergence of expertise level		Moses		Daphne	
	N	M	(SD)	M	(SD)
0	40	5307	(5447)	7065	(6828)
1	75	7159	(7965)	7627	(8875)
2	50	7340	(5908)	11208	(13538)
3	25	7660	(6821)	16898	(17666)

The distances for Moses were larger for higher divergence in expertise levels, but this tendency decreased for larger divergences. The distances for Daphne show constant increases of the distance with higher divergence of levels of expertise; thus, larger divergences in level of expertise are associated with an increase in distance.

Research question 4: Is a higher level of expertise associated with an increased number of fixated basic features?

**Figure 6.**
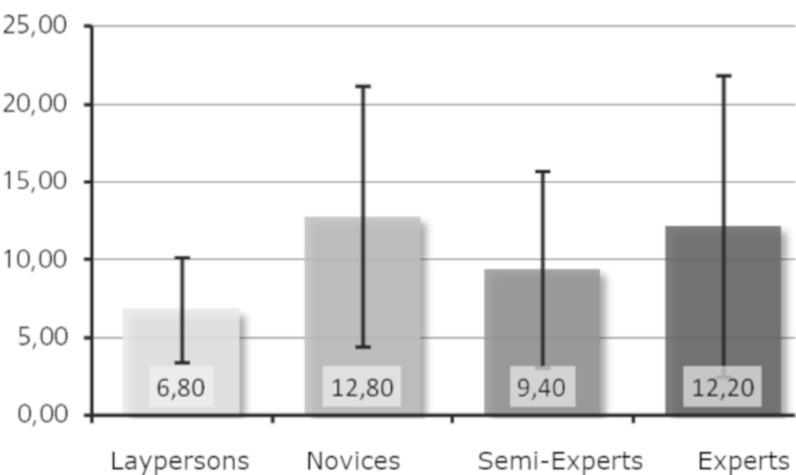
Number of fixated basic features (Moses).

**Figure 7.**
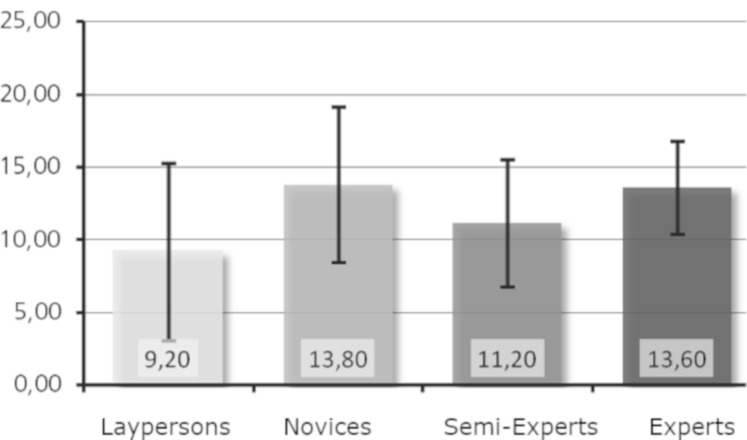
Number of fixated basic features (Daphne).

The mean values of the number of fixated basic features were very similar between groups of level of expertise (see Figure 6 and 7). In addition, the standard deviations within the groups were considerably large, as the box plots indicate. Research question 4 was therefore not supported by the data.

Research question 5: Is a higher level of expertise associated with an increased percentage of fixations on basic features?

**Figure 8.**
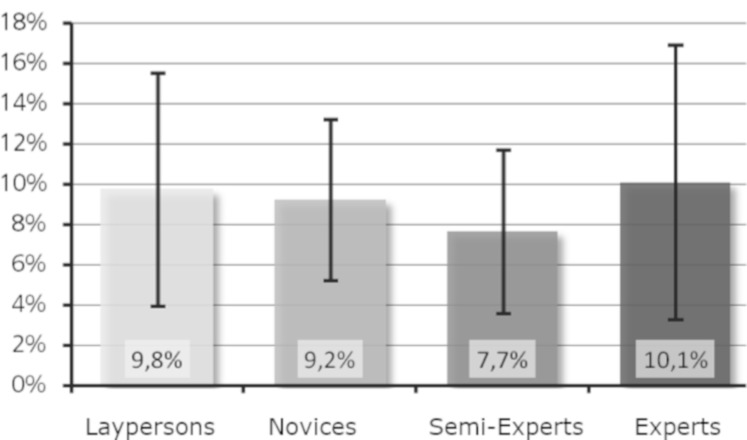
Percentage of fixations on basic features (Moses).

The mean values of the percentage of fixations on basic features for Moses (see Figure 8) showed the highest value for the experts, followed by the laypersons and then the novices. Proportionately, the semi-experts had the least fixations on the basic features of Moses.

**Figure 9.**
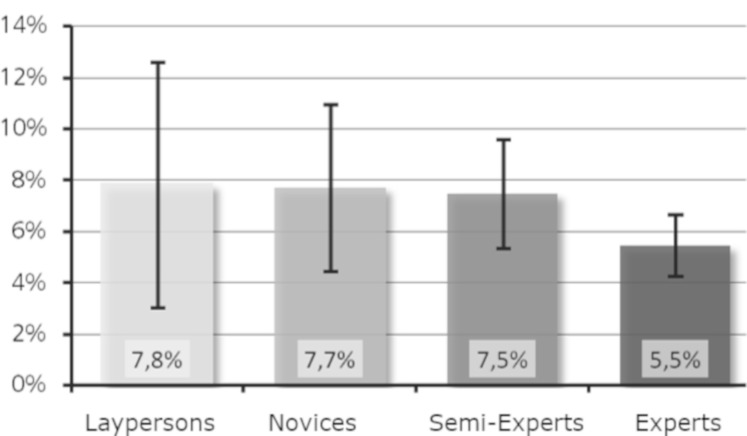
Percentage of fixations on basic features (Daphne).

For Daphne, on the contrary, the percentage of fixations on basic features descriptively decreased with increasing level of expertise (see Figure 9), although the differences were small. Taken together, the data did not support research question 5.

Research question 6: Is a higher level of expertise associated with an increased number of revisits per minute to basic features?

**Figure 10.**
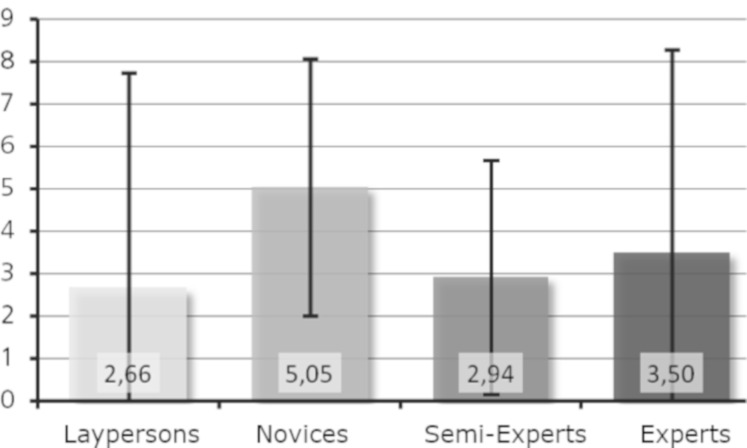
Revisits per minute on basic features (Moses).

**Figure 11.**
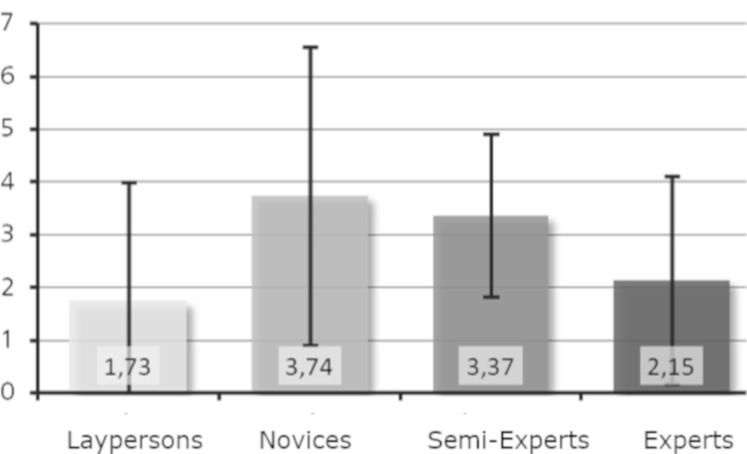
Revisits per minute on basic features (Daphne).

For both sculptures, the laypersons performed the least revisits, whereas the novices had the most revisits (see Figure 10 and 11). For Moses (Figure 10), the experts had more revisits than the semi-experts, whereas the opposite was true for Daphne (see Figure 11). When looking at the mean values of the groups, differences were visible. However, no general trend was observed concerning level of expertise; the curve is rather S-shaped for Moses but has an inverted U-shape for Daphne. We conclude that research question 6 has been partly supported.

## Discussion

The aim of this study was to establish how the eye movements of laypersons, novices, semi-experts and experts differ when looking at sculptures in real life. The results revealed some remarkable differences between the groups regarding the general inspection time, where experts are taking more time for the exploration.

Surprisingly, the results concerning global/local ratio (research question 1) were not in line with the results of prior research on two-dimensional artworks. In the present study, only small differences were found between the groups with respect to global/local ratio. This is in contradiction to Vogt and Magnussen (38) and Zangemeister et al. (44), who observed a higher global/local ratio among experts with two-dimensional stimuli. Both studies also distinguished between these two modes of perception based on saccade amplitude. In Nodine et al. (29) and in the present study, the distinction between global and local viewing was made according to fixation duration. Although a statistical relationship has been found between fixation duration and saccade amplitude (14), taking only one of these values into account allows situations in which fixations or saccades are incorrectly assigned to the global or local viewing mode. Looking at the other variable might provide clearer results by using the algorithm of Holmqvist and Andersson (14) that uses both fixation duration and saccade amplitude.

Here, an approach such as that of Fudali-Czyż et al. (9) may be of interest. In their study about the role of expertise in art on eye fixation-related potentials (EFRPs), they distinguished between ambient (global) and focal (local) modes by looking for a combination of short fixations followed by long saccades for ambient mode and long fixations followed by short saccades for focal mode. In the present study, however, due to the low sampling rate of 30 Hz, it was not possible to obtain meaningful data on saccades. The same difficulty occurs for the application of the local versus global scanpath analysis as proposed by Groner et al. (10) and Menz and Groner (26).

It is noticeable that the mean fixation time in the present study was lower than in studies with two-dimensional stimuli. Velichkovsky et al. (37), for example, used a threshold of 250 ms fixation time to distinguish global and local viewing. This threshold, however, was not feasible in our study, as some of the participants would no longer have had a single local fixation. Similarly, in Nodine et al. (29), only fixations longer than 400 ms were rated as local fixations.

The differences in fixation times between two- and three-dimensional stimuli are striking and require further attention to better understand the perception of three-dimensional works of art. Many perception models for viewing two-dimensional works of art are based on a two-phase process, in which distributed exploration is followed by specific analysis (1; 2; 4; 42). However, these models may be difficult to transfer to the reception of three-dimensional works of art such as sculptures. The “circumscribability” of a sculpture offers the viewer a wealth of new views from different observer perspectives. During the reception of a three-dimensional work, alternating processes of diversified and specific exploration can take place leading to a constant alternation between global and local processing. Although such a type of strategy was observed, no specific expertise-related differences were found.

In the present study, no consistent results were found concerning the switching between global and local viewing (research question 2). The frequency of the switching between global and local viewing has previously been examined by Nodine et al. (29), who found that experts switched less than laypersons between global and local viewing of the unchanged compositions, which the authors described as more balanced compositions. When considering modified (less balanced) compositions, however, the experts showed more frequent switching than the laypersons. Therefore, we expected to find differences in the switching between the global and local viewing as well. It is possible that either the appropriate operationalisation was not carried out, or the technical possibilities were not sufficient to adequately capture these differences in the present study.

The eyenalysis method (25) was used to compare the locations of fixations (research question 3). This method was extended so that all reference images of a sculpture could be included in the calculation of the distance of the gaze data of two participants. By including the three dimensions (x-coordinate, y-coordinate and fixation duration of each fixation), the eyenalysis method not only compared where the participants looked but also how long their gaze remained in a specific position. With this method, clear differences could be found in the distances, depending on whether the participant had the same or not the same expertise level. As the divergence in the expertise level increased, so did the distance between the visual data of the participants indicating that the gaze data for the laypersons were much more like the gaze data of other laypersons compared to the gaze data of the experts. The eyenalysis method may therefore reveal differences that are not present in other measures of the present study.

In the case of the number of fixated basic features (research question 4), the groups did not differ significantly. Looking at the mean values, it is noticeable that the order of the expert groups regarding the number of fixated basic features was similar for both sculptures: the laypersons fixated the fewest basic features, followed by the semi-experts and then the experts, while the novices fixated most of the basic features. The strongest outlier was an expert who, when looking at Daphne, fixated the most basic features of all (27 in total). However, no linear relation to the level of expertise was apparent. However, it is worth mentioning here that there are indications from other domains that the development of expertise is not linear, as it is for example stated by Lesgold et al. (23) in the domain of medicine.

When calculating the percentage of fixations on basic features (research question 5), the groups did not differ significantly either. Surprisingly, laypersons achieved high values here. With 9.8% (Moses) and 7.8% (Daphne), the laypersons had the largest and second largest percentage of fixations on basic features, respectively. This means that although they fixated fewer basic features overall (average of 9.2), they fixated more on these few basic features than the other groups. These results contrast with the experts, who fixated an average of 13.6 basic features for Daphne and with a percentage of fixations on basic features of only 5.5%. Although the experts fixated basic features proportionally less often, they fixated more of them than the laypersons. These results can be interpreted as a more global approach and faster processing of the basic features by the experts. Efficient perception acquired through training shortens the time required for information processing and thus enables an extension of the processing time. The results from Velichkovsky et al. (37) about automation of skills may also suggest that experts are able to capture details in the global viewing mode for which the laypersons or novices must utilize the local viewing mode. Future investigations will be necessary to investigate this effect, for example, with short secondary tasks, like search or remembering comparing experts and laypersons.

Regarding research question 6, the laypersons had the minimum number of revisits per minute for both sculptures. A revisit was only counted if the basic feature was left (i.e., a fixation outside the area of interest), and a next fixation was made inside the area of interest again. Here the laypersons fixated longer the basic features and made several fixations within these basic features, returning to them less often, indicating an investigation of relationships between the basic features and a comparison between basic features and the whole sculpture. Surprisingly, the experts also had only a few revisits per minute. The largest number of revisits per minute was shown for both sculptures by the novices still in training.

As a consequence of these results future research should redefine the operational definitions of global and local viewing following the proposals and definitions and identification of basic features by Groner et al. (10), Menz and Groner (26), Kołodziej et al. (20), Hein and Zangemeister (11). For further research, machine learning could be used to reduce the time-consuming mapping of AOIs (41). Although the eyenalysis method (25) has the potential to uncover relevant differences, thus far we have only a few indications of what form these differences take. The fact that experts and novices view artworks differently has been shown repeatedly for two-dimensional works, and attempts have even been made to identify experts in the visual arts based on the oculographic data when viewing paintings (8; 20). In the three-dimensional domain of sculpting, by contrast, such information is clearly lacking.

A challenge to the present study was its three-dimensionality and the calculation over eight different reference images. Mobile eye tracking in real-life settings automatically poses a problem when evaluating fixations to a three-dimensional stimulus: the individual fixations from the eye movement video must be transferred to a common reference via a mapping procedure or via manual coding within an evaluation programme in order to be able to compare the sets of eye movement data. By default, a two-dimensional photo is used for this purpose. The three-dimensionality of the object is disregarded or reduced to a two-dimensional image. In this case, however, information is lost. In many domains, such as medicine, car-driving, or aircraft, research is done in real life settings and three-dimensional stimuli are used, but when it comes to evaluating the data, a two-dimensional reference is used again. Moreover, in some studies the actual three-dimensional stimuli are displayed on a monitor or as a simulation. Same is true for the domain of sculpture. Sculptures have been used as stimuli in some studies, but these were always only available in two-dimensional form (photo/monitor). In addition, only one view of the sculpture was presented, e.g., a bust in profile.

To address this discrepancy is especially important for eye movement studies in the domain of sculpting. In the future, it would be desirable to embed a sculpture as a 3D model into the eye tracking evaluation programme. Such an approach would allow mapping the data directly to a three-dimensional reference model rather than eight individual reference images. Eye movements that change from one perspective to the next could thus be better understood. A transfer of methodology from other application areas of eye tracking, like 3D geo-visualization (13) seems appropriate. Currently, we are working on a photogrammetry-based solution for an automatised 3D mobile eye tracking mapping tool.

Our study was exploratory in nature and an attempt to address the joint analysis of eye movements and levels of expertise in authentic settings. It can be seen as a starting point to tackle the question of how to sensibly operationalise “level of expertise” in such settings. Certainly, this exploratory character comes with several limitations (22) that need to be addressed in future research. Although the sampling in our study was theoretically founded, the grouping of the levels of expertise might not necessarily be the best. Due to technical problems, we also had to exclude a high number of potential participants. Likewise, although we strived for a highly ecologically valid environment, this authentic environment also defined sculptures and procedures. Therefore, the results may be artefacts of the sculptures found in the museum and the exhibition characteristics. The study has shown that the selection of the stimulus, as well as the local conditions can have a great influence on the results. For example, the sculpture must be large enough to ensure sufficiently accurate resolution with the eye tracker. However, if the sculpture is too large, the risk increases that the eyes of the subjects leave the detection range of the eye tracker, e.g., when looking extremely upwards. In the present study, the small size of the sculpture Moses made the mapping process significantly more difficult and could thus have an impact on accuracy. Regarding the local conditions, the positioning of the sculpture Daphne in the middle of an otherwise empty room (no other sculptures in the room) proved to be very positive. This was not the fact for the sculpture Moses due to other sculptures in the vicinity. The way artworks are displayed is known to have an impact on the way a museum visitor views them (33). These influencing factors could be remedied by means of a laboratory situation. However, a lab could impact the natural observation situation and thus change the participants’ behaviour. In our study, the selection of sculptures was limited by the current exhibition in the museum and the focus of the study was the exploration in a natural setting. These considerations outweighed all other concerns.

To draw conclusions about the influence of different design styles on the reception of the artworks, further research with additional stimuli needs to be done. Experimental consolidation is needed to further investigate the eye movements and information processing of three-dimensional visual art objects. Another artefact that may have occurred is the specification of basic features. These were defined by only one expert, and further validation would be desirable. For the analysis, eyenalysis was used; as an experimental methodology, it is potentially powerful but in need of more testing and validity checks.

In conclusion, the measures applied in this study were reasonable and sensible – if they do not meet theoretical predictions, this might also cast doubt on some of them. We need to be cautious when applying theoretical assumptions from other professional domains, as they may not easily be transferred to authentic vision processes in visual arts such as sculpture.

Although this study was set up very thoroughly and investigated participants’ behaviour systematically, it was not possible to distil a clear feature of visual expertise in this way. Therefore, future research needs to dig deeper and keep on studying this intriguing topic.

### Ethics and Conflict of Interest

The authors declare that the contents of the article are in agreement with the ethics described in http://biblio.unibe.ch/portale/elibrary/BOP/jemr/ethics.html and that there is no conflict of interest regarding the publication of this paper.

### Acknowledgements

We wish to thank the museum Ostdeutsche Galerie Regensburg for providing the room and the artworks. Moreover, a special thanks is dedicated to the artists and all other persons who took their time to participate in the study and those who provided valuable advice throughout the project.

### Artworks/Stimuli

Artworks/Stimuli

Kroner, Kurt (1919). ‘Untitled (Moses)’, [Ohne Titel (Moses)], bronze, 38,9 x 17,2 x 19,9 cm, Kunstforum Ostdeutsche Galerie Regensburg, Inv.-Nr. 3881, Loan from the Bundesrepublik Deutschland.

Sintenis, Renée (1930 draft version / 1991 cast, posthum) ‘Great Daphne’ [Große Daphne], bronze on pedestal of dark grey shell lime-stone, 144 x 29,5 x 25,5 cm, Kunstforum Ostdeutsche Galerie Regensburg, Inv.-Nr. 18710, Loan from the Bundesrepublik Deutschland, © VG Bild-Kunst, Bonn.
